# Supplementary material for: Training providers to implement heart failure shared medical appointments: A qualitative evaluation
Source: PLoS One. 2024 Nov 20;19(11):e0310639. doi: 10.1371/journal.pone.0310639 (PMC11578495; doi:10.1371/journal.pone.0310639)
Supplement: S1 Appendix — (PDF) [file pone.0310639.s001.pdf]

19028 - H01 8765

S1: Pre-training IG

HF-SMA Implementation *Pre-Implementation* Interview Guide
(Leadership, Providers, Administrative Staff)

Interviewer Name:

Note taker Name:

Interviewee:
Site:

Date:

Time Start:
Time End:

My name is [interviewer name] and joining me is my colleague [note taker name] who will be taking notes. We are studying the implementation of Shared Medical Appointments for heart failure (HF-SMA) as part of an HSRD funded implementation study.

Our findings will be summarized in a report to HSRD and to our central office partners, the Office of VA Access to Care. We won't identify you in any of our reports or publications and your responses will not be shared in an identifiable format. We will combine feedback from other participants whenever possible and will share specific suggestions without referencing the person who said them. Your responses will be kept anonymous and confidential.

The call will take approximately 20-30 minutes, and someone may request to speak with you again in approximately one year.

Your participation in this interview is voluntary. You can stop the interview at any time and let us know if you'd rather not answer a particular question, because you can always skip it.

Do you have any questions?

In order to make sure we capture all of the information you give us, we would like to record this call. The audio-file for the recording will be stored directly to restricted access file on the VA intranet. Is this okay with you? **[Hit record button.]** Okay, to confirm, I'm starting the recording. Is this ok with you? If no: Would you like to continue the interview and I will take notes?

[Generic prompts: If responses are limited or require clarification, probes may be used to illicit more detailed responses. Probes should use words or phrases presented by the participant using one of the following formats:
1. What do you mean by ___________ ?
2. Can you tell me more about ___________ ?
3. Can you give me an example of ___________ ?
4. Can you tell me about a time when ___________ ?]

APPROVED
1 / 10 / 20
VANEOHS
Institutional Review Board

1. Please tell us about your experience with Heart Failure Shared Medical Appointments at your site?

2. What is your opinion of the evidence supporting the use of shared medical appointments?

3. How do HF SMAs align with your facility's goals,
   a. priorities, (provide example of priorities)
   b. and patient populations?

4. How do you expect the use of HF SMAs will or could affect?
   a. wait times
   b. patient care
   c. Clinic utilization

5. What resources will be needed to support for implementation of HF SMAs?
   i.e. space
   i.e. scheduling support
   i.e. time

6. What challenges do you foresee implementing HF SMAs at your site?

7. What could help facilitate implementation of HF SMAs at your site?

8. Is there anything else you think would be valuable for us to know about your site's ability to incorporate it successfully Heart Failure Shared Medical Appointments? *
