# Supplementary material for: Training providers to implement heart failure shared medical appointments: A qualitative evaluation
Source: PLoS One. 2024 Nov 20;19(11):e0310639. doi: 10.1371/journal.pone.0310639 (PMC11578495; doi:10.1371/journal.pone.0310639)
Supplement: S2 Appendix — (PDF) [file pone.0310639.s002.pdf]

HF-SMA Implementation *Post-Training* Interview Guide
(Providers and Administrative Staff)

Interviewer Name:

Note taker Name:

Interviewee:
Site:

Date:

Time Start:
Time End:

Hello [Dr./Mr.Ms. interview participant name],

My name is [interviewer name] and joining me is my colleague [note taker name] who will be taking notes. We are studying the implementation of shared medical appointments for heart failure. Today we are interested in learning about the effectiveness of the training program. Your responses will be used to improve future programs.

Our findings will be summarized in reports to HSRD and the Office of VA Access to Care. We won't identify you in any of our reports or publications and your responses will not be shared in an identifiable format. We will combine feedback from other participants whenever possible and will share specific suggestions without referencing the person who said them.

The call will take approximately 30 minutes.

Your participation in this interview is voluntary and your responses will be kept anonymous and confidential. You can stop the interview at any time and let us know if you'd rather not answer a particular question.

Do you have any questions?

In order to make sure we capture all of the information you give us, we would like to record this call. The audio-file for the recording will be stored directly to restricted access file on the VA intranet. Is this okay with you? **[Hit record button.]** Okay, to confirm, I'm starting the recording. Is this ok with you? If no: Would you like to continue the interview and I will take notes?

[Generic prompts: If responses are limited or require clarification, probes may be used to illicit more detailed responses. Probes should use words or phrases presented by the participant using one of the following formats:
1. What do you mean by ____________ ?
2. Can you tell me more about ____________ ?
3. Can you give me an example of ____________ ?
4. Can you tell me about a time when ____________ ?]

APPROVED
1 / 10 / 20
VANEOHS
Institutional Review Board

1. Which training method did you receive regarding Heart Failure Shared Medical Appointments training program, in-person or video-assisted?
    a. In-person
    b. Video assisted

2. How did you learn about this opportunity?

3. How did you decide to attend this training?

4. Tell us about your general experience as a participant in the Heart Failure Shared Medical Appointments training program. *.

Initial Setup
First, we are interested in learning how this training prepared you to work with others at your facility on initial setup of the shared medical appointments.

5. How prepared are you to initiate shared medical appointments at your site?

6. What parts of the training were most helpful for initial set up? In what ways? (use probes)
    a. Which topics?
    b. Which activities?
    c. What was not helpful?
    d. What suggestions do you have for improving the training?

7. What other resources, knowledge or support will you need before initiating shared medical appointments at your site

8. To what extent did this training address barriers to implementation at your site?

Establishing a curriculum
Next, we are interested in learning about the usefulness of the sessions on specific knowledge related to heart failure management.

9. From your perspective, tell us about the usefulness of the information related to heart failure self-management presented at the training.
    a. Which topics were most helpful/ least helpful?
    b. Which activities were most helpful/ least helpful??
    c. What suggestions do you have for improving the training?

10. How confident are you in creating your own curriculum for shared medical appointments after attending this training? (probe)

Format
Next, we are interested in learning about the format of the training and in particular your experience attending the in-person/virtual training.

11. What aspects of the (in-person/virtual) format were most helpful/valuable? *
    a. What was least helpful/valuable?
    b. How would you improve?

c. What aspects worked best?
d. What did not work as well?
   Do you feel that you have a connection with the facilitators?
e. Did the training provide a level of confidence for its implementation, if so how?

Leaders.
We want to learn about the effectiveness of the trainers.

12. To what extent were the trainers knowledgeable about heart failure?

13. To what extent were the trainers knowledgeable about setting up shared medical appointments for heart failure?

14. To what extent were the trainers knowledgeable about leading shared medical appointments for heart failure?

Tacit knowledge/Mentorship
We have just a few more questions.

15. What are the intangible benefits to your participation in this training? i.e. decision making?

16. How has your working relationship with others at your site changed as a result of participation in this training?

17. How has participation in this training affected your understanding of other roles and responsibilities in setting up, creating and leading shared medical appointments?

18. How likely are you to contact your trainers now that you have finished this training?

19. Is there anything else you think would be valuable for us to know about Heart Failure Shared Medical Appointments training program or your ability to incorporate it successfully? *

Thank you for your time.
