# Supplementary material for: Training providers to implement heart failure shared medical appointments: A qualitative evaluation
Source: PLoS One. 2024 Nov 20;19(11):e0310639. doi: 10.1371/journal.pone.0310639 (PMC11578495; doi:10.1371/journal.pone.0310639)
Supplement: Attachment — Submitted filename: Response to Reviewers 2.docx [file pone.0310639.s004.docx]

March 4, 2024

Edwin Ularte

PLOS ONE

Re:	PONE-D-24-07219

Thank you for the review of our manuscript.

We addressed the three items listed on as follows:

(1)We noted a different IRB approval number (1583993) in your online submission form which does not match with the approval document (Study CY19-028). To proceed with the manuscript, we request you to provide the correct IRB document or update the correct IRB approval number in the online submission form. Please note that some fields in the submission form may reset with this change, so please go through your submission in full to ensure that all information is accurate and complete when resubmitting your manuscript.

The IRB approval number is different due to the change in systems utilized during the project. The original number is CY 19-028. I have corrected the IRB number to align with the original system.

(2)The individual David Aron named as investigator on the ethics documents is not an author on the present submission. As we would generally expect the investigator named on the ethical approval to be an author on the submission, can you please provide an explanation for this situation?

Dr. Aron retired and Dr. Ball became PI. Dr. Aron is listed in the acknowledgement section for his role within the project.

We have included the approval of the change in PI. The file name is 7-19028-H01 Amendment approval.pdf; however, in the body of the approval it references IRBNet# 1583993-7 in the memo portion. If you scroll to the bottom, you will see a mixture of both numbers in the Documents Reviewed section.

(3)We have noticed that you have provided two dates for recruitment (see lines 34-35 and 53-55). Please clarify and ensure that you report in the Methods section the day, month and year of the start and end of the recruitment period for this study (recruitment refers to when the participants were formally enrolled into the study). Please note that if this information is not included when your manuscript is resubmitted, it may be rejected. Please advise if we missed any requested item. Thank you.

The site recruitment (lines 134-135) is different from the interview recruitment of the recruited training sites (lines 153-156). Clarification of this has been added.

Respectfully,

Rene Hearns
